# Quantitation of the latent HIV-1 reservoir from the sequence diversity in viral outgrowth assays

**DOI:** 10.1186/s12977-018-0426-1

**Published:** 2018-07-05

**Authors:** Art F. Y. Poon, Jessica L. Prodger, Briana A. Lynch, Jun Lai, Steven J. Reynolds, Jingo Kasule, Adam A. Capoferri, Susanna L. Lamers, Christopher W. Rodriguez, Daniel Bruno, Stephen F. Porcella, Craig Martens, Thomas C. Quinn, Andrew D. Redd

**Affiliations:** 1 Department of Pathology and Laboratory Medicine, Western University, London, ON Canada; 2 Department of Microbiology and Immunology, Western University, London, ON Canada; 3 Laboratory of Immunoregulation, National Institute of Allergy and Infectious Diseases, National Institutes of Health, Baltimore, MD USA; 4 Department of Medicine, Johns Hopkins School of Medicine, Baltimore, MD USA; 5 Rakai Health Sciences Program, Kalisizo, Uganda; 6 BioInfoExperts, LLC, Thibodaux, LA USA; 7 Genomics Unit, Research Technologies Branch, Rocky Mountain Laboratories, National Institute of Allergy and Infectious Diseases, National Institutes of Health, Hamilton, MT USA

**Keywords:** Latent viral reservoir, HIV-1, Viral outgrowth assay, Limiting dilution assay, Next-generation sequencing, Bayesian inference

## Abstract

**Background:**

The ability of HIV-1 to integrate into the genomes of quiescent host immune cells, establishing a long-lived latent viral reservoir (LVR), is the primary obstacle to curing these infections. Quantitative viral outgrowth assays (QVOAs) are the gold standard for estimating the size of the replication-competent HIV-1 LVR, measured by the number of infectious units per million (IUPM) cells. QVOAs are time-consuming because they rely on culturing replicate wells to amplify the production of virus antigen or nucleic acid to reproducibly detectable levels. Sequence analysis can reduce the required number of culture wells because the virus genetic diversity within the LVR provides an internal replication and dilution series. Here we develop a Bayesian method to jointly estimate the IUPM and variant frequencies (a measure of clonality) from the sequence diversity of QVOAs.

**Results:**

Using simulation experiments, we find our Bayesian approach confers significantly greater accuracy over current methods to estimate the IUPM, particularly for reduced numbers of QVOA replicates and/or increasing actual IUPM. Furthermore, we determine that the improvement in accuracy is greater with increasing genetic diversity in the sample population. We contrast results of these different methods applied to new HIV-1 sequence data derived from QVOAs from two individuals with suppressed viral loads from the Rakai Health Sciences Program in Uganda.

**Conclusions:**

Utilizing sequence variation has the additional benefit of providing information on the contribution of clonality of the LVR, where high clonality (the predominance of a single genetic variant) suggests a role for cell division in the long-term persistence of the reservoir. In addition, our Bayesian approach can be adapted to other limiting dilution assays where positive outcomes can be partitioned by their genetic heterogeneity, such as immune cell populations and other viruses.

**Electronic supplementary material:**

The online version of this article (10.1186/s12977-018-0426-1) contains supplementary material, which is available to authorized users.

## Background

**Fig. 1:**
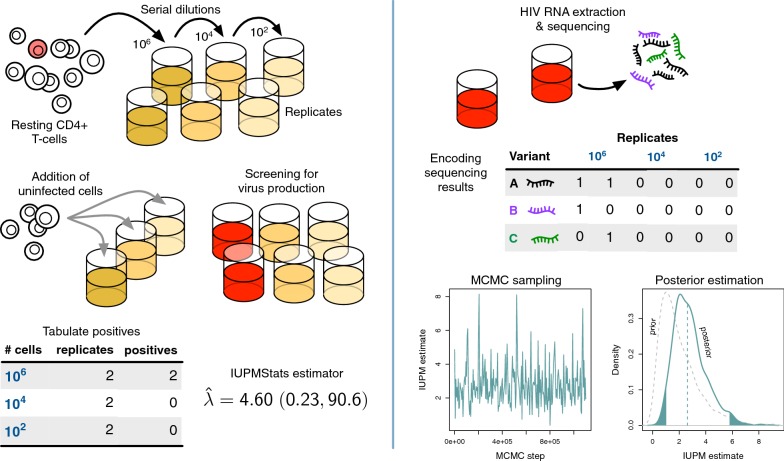
Schematic diagrams of experimental and data analysis procedures. (Left panel) Resting CD4+ T cells sampled from an HIV+ patient are serially diluted in replicate culture wells. Uninfected cells are added to the culture wells to amplify viral outgrowth (red). IUPMStats estimates the rate parameter $\hat{\lambda }$ of the single-hit Poisson model from the numbers of positive wells at varying dilutions. (Right panel) HIV-1 RNA is extracted from each positive well and amplified for library construction and sequencing. The presence/absence of different sequence variants are tabulated and used to fit a multi-target Poisson model by Markov chain Monte Carlo (MCMC) sampling, in which a prior distribution on the IUPM (lower right, grey dashed curve) is updated by the data to estimate the posterior distribution (solid curve)

HIV-1 persists in individuals, despite fully suppressive anti-retroviral therapy (ART), due to the presence of resting CD4+ (rCD4) T cells that are latently infected with replication competent, integrated copies of the virus [1]. These resting cells can reactivate through natural immunological challenge within the body, and upon reactivation will produce progeny virus. In the case of discontinuation of ART, this reactivation will lead to full viral rebound within a matter of weeks in virtually all individuals [2]. The size of this pool of latently infected cells, referred to as the latent viral reservoir (LVR), has proven difficult to measure as the vast majority of integrated proviral DNA is defective [3, 4]. Therefore current methods for quantifying replication-competent provirus rely on the production of infectious virus in vitro; the quantitative viral outgrowth assay (QVOA) is presently the gold standard for LVR quantification. The QVOA is based on the in vitro reactivation of rCD4 T cells isolated from an infected individual in a limiting dilution of replicate wells, with a readout of HIV-1 p24 antigen production to identify wells containing at least one latently infected cell. However, the QVOA is labor-intensive and difficult to scale up to larger numbers of wells. Each well is cultured for several weeks with the repeated addition of uninfected CD8-depleted lymphoblasts, or the single addition of MOLT-4/CCR5 cells, to amplify any viruses released so that HIV p24 antigen can be produced at detectable levels [5, 6] (Fig. 1). Consequently, it would be beneficial to minimize the number of replicate wells that need to be cultivated for every patient sample. Sequencing individual viruses from the positive wells internally dilutes each virus population by partitioning the binary outcome (p24 antigen positive) into multinomial outcomes (presence or absence of sequence variants) [7]. In other words, the genetic diversity of the virus population provides an intrinsic replication and dilution series that can enable the investigator to culture a smaller number of wells, and has therefore been proposed as a tool for refining the estimates of infectious units per million (IUPM) cells [7], the standard LVR measure.

Additionally, sequencing of outgrowth virus can provide information on the contribution of rCD4 T cell division to persistence of the LVR. The long half-life of the HIV-1 LVR that averages several years in duration [8, 9], in combination with the presence of clonal populations of rCD4 T-cells that contain identical HIV-1 proviral sequences [10–12], suggests that rCD4 T-cell proliferation may play a significant role in maintaining the LVR. The role that this phenomenon plays in maintaining the LVR is of critical importance for HIV-1 cure efforts, as it will help direct the ideal path for targeting and eventually destroying these latently infected cells. To this end, multiple groups have begun using viral sequencing of both latent HIV-1 proviral DNA, as well as populations derived from viral outgrowth assays (such as the quantitative viral outgrowth assay [QVOA]), to characterize the contribution of this clonal expansion to the maintenance of the LVR [10, 13–15].

### The single-hit Poisson model

The current standard approach for estimating the IUPM from QVOA results is based on the single-hit Poisson model, which has long been used for the analysis of limiting dilution assays [16]. The expected IUPM is estimated by maximizing the likelihood for a binomial distribution:

1 $$\begin{aligned} L(Y|\lambda ,N) \propto p_\lambda ^Y (1-p_\lambda )^{N-Y} \end{aligned}$$

where *N* is the number of wells in the QVOA; *Y* is the number of p24-positive wells; and $p_\lambda =1-\exp (-n\lambda )$ is the probability that a well is positive because one or more of the cells within it were infected (hence ‘single-hit’), given that each well contained *n* cells. The rate parameter $\lambda$ is the proportion of cells that are infected, from which we derive our estimate of the IUPM as $\lambda \times 10^{6}$. This model assumes that all cells in the sample population have a uniform probability of being infected. It is conventionally assumed that *n* (the number of cells per well) is known without error, so that the maximum likelihood estimate (MLE) of $p_\lambda$ is a direct estimate of $\lambda$. Serial dilution is typically used to vary *n* across sets of wells, which reduces the chance that the wells are either all positive or all negative; neither of these extreme outcomes can be used effectively to estimate $\lambda$. Thus, a more general formulation of equation (1) is:

2 $$\begin{aligned} L(Y|\lambda , N) = \prod _{i=1}^{D} \left( 1-\exp (n_i \lambda )\right) ^{Y_i} \exp (n_i \lambda )^{N_i-Y_i} \end{aligned}$$

where *D* is the number of dilution factors, $N_i$ is the number of wells with the *i*-th dilution factor, $Y_i$ is the number of these wells that test positive, and $n_i$ is the number of cells per well [17].

### Incorporating sequence variation

The defining feature of the single-hit Poisson (SHP) model is that each well has a binary all-or-nothing outcome, in which the well either tests positive or negative for viral outgrowth (Fig. 1). Estimating IUPM is contingent on having a mix of positive and negative outcomes, where the probability of each positive outcome (that a well contains at least one infected cell, and therefore p24 is detected) is modulated by dilution. However, if we obtain genetic sequences for individual viruses within a given well, then we have additional information about the number of infected cells per well [7]. If two distinct sequence variants are observed from a well, for instance, then we can assume that the well initially contained at least two infected cells. Additionally, we can track the occurrence of specific variants across wells to simultaneously estimate the relative frequency of variants in the sample population of infected cells, which also provides information about the clonal expansion of infected cells. For example, consider a QVOA where four wells are plated with $10^6$ cells per well, and p24 ELISA shows that all four wells are positive for HIV. From these data, there is no sensible maximum likelihood estimate for $\lambda$ under the SHP model because the likelihood surface asymptotes as $\lambda$ approaches infinity. However, suppose there are five genetic variants (labeled *A* through *E*) with a uniform frequency of 0.2 in the sample population of viruses, and the true IUPM is 5. Then we can simulate a random outcome represented by the following binary (presence/absence) matrix:

$$\begin{aligned} \begin{array}{llccccc} {\text{Well}} &{} {\text{p24}} &{} A &{} B &{} C &{} D &{} E\\ \hline 1 &{} + &{} 0 &{} 1 &{} 0 &{} 1 &{} 0\\ 2 &{} + &{} 1 &{} 0 &{} 0 &{} 0 &{} 0\\ 3 &{} + &{} 1 &{} 1 &{} 0 &{} 0 &{} 0\\ 4 &{} + &{} 0 &{} 1 &{} 1 &{} 1 &{} 0\\ \end{array} \end{aligned}$$

Under these conditions, the probability that all four wells are positive is $(1-e^{-5})^4\approx 0.973$. In other words, there is a good chance that we will be unable to estimate the IUPM with the SHP model. On the other hand, we expect that each variant will be present in a given well only $1-e^{-0.2\times 5} \approx 0.63$ of the time, and all four wells only about $0.63^4 \approx 16\%$ of the time; the chance that all variants appear in all wells is only about one-hundredth of a percent. Therefore, if we use sequencing to determine which variants appear in each well, then it is very likely that we can regain the ability to estimate the IUPM in an ‘all positive’ scenario. Incorporating sequence information constitutes a more difficult problem because we need to simultaneously estimate the variant frequencies along with $\lambda$.

Recently, Lee et al. [7] described using a primer ID-based next-generation sequencing assay (NGSA) to sequence the virus populations in positive QVOA wells. This QVOA-NGSA method employs a maximum likelihood estimator for IUPM that is the sum of variant-specific quantities, $\hat{\lambda } = \sum _i -\log (1-y_i/K)$, where $y_i$ is the number of *K* wells containing the *i*-th variant. This estimator stipulates that every well contains the same number of cells, such that the estimate $\hat{\lambda }$ can be rescaled by a constant factor to estimate the IUPM; for instance, if each well contained $2.5\times 10^6$ cells then ${\text{IUPM}} = \hat{\lambda } / 2.5$. Furthermore, this estimator has the same problem as the SHP model in that $\hat{\lambda }$ goes to $\infty$ if any variant is observed in every well ($y_i=K$). This potentially limits the utility of the estimator when the number of wells is small, the number of cells per well is large, or the actual IUPM is large and/or predominated by a single variant (high clonality) [14].

In this study, we developed a Bayesian method (IUPMBayes; Fig. 1) to jointly estimate the IUPM and the frequency distribution of variants from next-generation sequence data obtained from the positive wells of viral outgrowth assays. We perform an extensive simulation study to compare our Bayesian method to the maximum likelihood estimators developed for the SHP model [IUPMStats; 17] and the QVOA-NGSA method [7]. Lastly, we compare the performance of these methods using next-generation sequencing of two genetic regions (*pol* and *gp41*) of viral outgrowth populations from positive wells, derived from peripheral blood samples from two ART-suppressed individuals with markedly different levels of HIV sequence diversity in the LVR.

## Results

### Simulation results

**Fig. 2:**
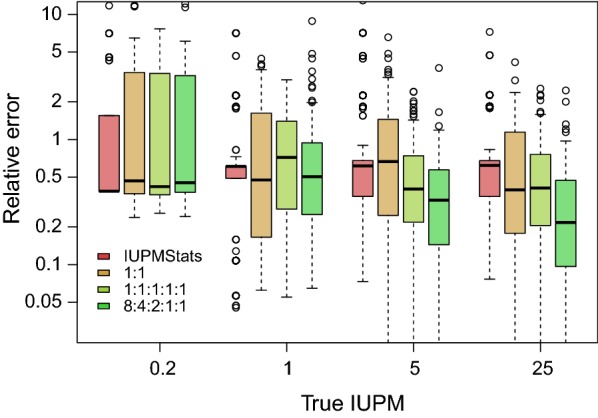
Relative error in estimating IUPM for data simulated under the standard experimental design. We calculated the relative error of an estimate $\hat{x}$ given true value *x* as $|(\hat{x}-x)/x|$. Each set of box-and-whisker plots summarizes the relative errors for estimates obtained by IUPMStats (red) and IUPMBayes under three different sets of variant frequencies (see inset legend) for a given true value of IUPM (0.2, 1, 5 and 25 per million cells). We used a log-transformation of relative errors and rescaled the *y*-axis to clarify differences between methods and simulation conditions; 25 outliers with relative errors below .03 were excluded from this plot region

We simulated data sets under an experimental design in which 12 wells were inoculated with serial dilutions of $10^6$, $2\times 10^5$, $4\times 10^4$, 8000, 1600 and 320 cells per well in duplicate. This design was configured to be representative of viral outgrowth assays used in previous empirical studies [6, 8]. Next, we used these simulations to evaluate the accuracy of IUPMStats, which combines a maximum likelihood estimator on the single-hit Poisson model with a Bayesian estimator for cases where no positive wells were observed, and our Bayesian method (IUPMBayes) that utilizes sequence variation in addition to the number of positive wells. For these experiments, we used the uninformative prior distribution on $\lambda \sim U(0,\infty )$. Our results are summarized in Fig. 2. First, we observed that the relative errors associated with estimates from IUPMStats were relatively consistent across the IUPM values used to simulate the data sets, with an overall median relative error of about 0.49. When the true IUPM was low (0.2 per million cells), then similar estimates were obtained using either method. However, the overall relative error decreased significantly with increasing IUPM (log-link gamma generalized linear model, GLM; $t=-\,9.0$, $P < 10^{-15}$). If we assumed that the sample population contained only two variants at equal frequencies (1:1), then we observed no significant improvement in IUPMBayes above IUPMStats (paired *t*-test, $t=-1.05$, $P=0.29$). However, IUPMBayes gained a significant advantage with increasing virus sequence diversity and IUPM (interaction term $t=-4.5$, $P=6.0\times 10^{-6}$; Fig. 2), where we converted the variant frequency distributions into Shannon entropy values to quantify diversity [18]. Adding this interaction term to the gamma GLM conferred a significant improvement in fit (likelihood ratio test, LRT; ${\text{df}}=2$, $P=1.8\times 10^{-5}$).

**Fig. 3:**
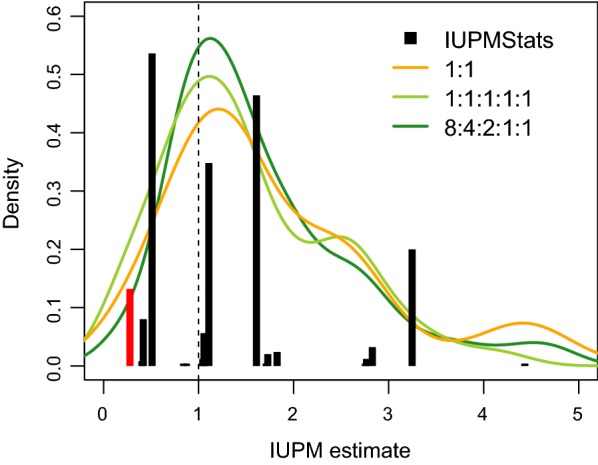
Distribution of estimates from IUPMStats and IUPMBayes for simulations given IUPM = 1. We used a barplot to summarize the distribution of IUPMStats estimates, which makes clear that these estimates are limited to a relatively small number of values given the dilution series and number of replicate wells in our simulation experiments. The leftmost bar (red) corresponds to the median Bayesian posterior estimate employed by IUPMStats when all wells are negative. The distributions of estimates from IUPMBayes under two sets of variant frequencies (1:1 and 1:1:1:1:1) are summarized with Gaussian kernel densities (curves). Unlike the IUPMStats estimates, these distributions were unimodal and centred near the true value. We obtained similar results under varying conditions and IUPM values

Why do we observe significant differences in accuracy between IUPMStats and our Bayesian method? First, the maximum likelihood estimator (MLE) used in the IUPMStats can only assume a finite number of values because the outcome space is discrete. When there are a large number of wells in which a positive outcome is likely to occur, then there is a high probability that the true value is close to the MLE. As the expected number of positive wells declines, however, the distribution of potential MLEs becomes increasingly sparse. For example, the most frequent IUPMStats estimates when the true IUPM was set to 1 were 0.51 and 1.61 (Fig. 3), which correspond respectively to the outcomes where one or both of the wells with $10^6$ cells plated were positive. The next two most frequent MLEs corresponded to the outcomes where both $10^6$ wells were positive and either one or both of the wells with $2\times 10^{5}$ cells were positive. In contrast, a Bayesian approach attempts to generate a random sample from a continuous posterior distribution over the IUPM parameter.

Second, the wells with high cell counts become less informative with increasing IUPM, since they will nearly always be positive when the IUPM is sufficiently high. For example, a well with $10^6$ cells has a probability $1-\exp (-5)=0.993$ of being positive when the IUPM is 5. Sequencing the outgrowth variants in positive wells can restore the information content of these wells, because these additional data partition the outcomes into variant-defined types. Thus, the accuracy of IUPMBayes improves with increasing IUPM but only when the sequence variants are present at informative frequencies. For instance, the advantage of utilizing sequence information deteriorates as the frequency distribution is skewed toward a single predominant variant (Additional file 1: Fig. S1), which also reduces the Shannon entropy. In this extreme case, most of the variants are so rare that they are seldom sampled in the positive wells.

### Reducing the number of wells

**Fig. 4:**
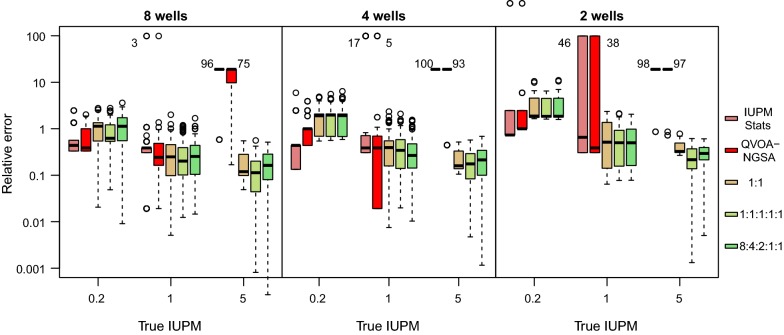
Relative errors in IUPM estimates for simulated data with uniform cell counts. Each box plot summarizes the distribution of relative error for 100 replicate simulations for the respective methods: IUPMStats, QVOA-NGSA, and IUPMBayes (three sets of variant frequencies, see legend). The plots are drawn on a log-transformed *y*-axis to facilitate comparison between methods under varying conditions. When the maximum likelihood estimators employed by IUPMStats or QVOA-NGSA were unable to produce a finite estimate of IUPM, we assigned an arbitrary value of 100 cells per million. The respective box plots are directly labeled with the numbers of non-finite estimates at the outliers or median bands

We conducted an additional set of simulation experiments in which the same number of cells ($10^6$) were plated in replicate wells. This configuration facilitated the comparison of our method to the QVOA-NGSA maximum likelihood estimator proposed by Lee et al. [7], which requires that the number of cells is constant across wells. Moreover, we used these experiments to evaluate the sensitivity of the methods to reducing the total number of wells to 8, 4 and 2 per experiment. Given the sparseness of these data, we employed an informative prior distribution on $\lambda _m\sim \Gamma (\alpha =2, \beta =1)$. Our results are summarized in Fig. 4. First, we observed that IUPMBayes tended to overestimate the IUPM relative to the other methods when the true value was 0.2, where full sets of negative wells were common. The median relative error was 1.09, which corresponds to an overestimate of about 0.37 cells per million. When the IUPM is set this low, many replicate experiments will result in all-negative outcomes with probability $\exp (-0.2\times n)$, where *n* is the total number of replicates. This lack of information in turn results in greater uncertainty in estimating IUPM. For this situation, IUPMStats uses the posterior median estimate assuming a uniform prior on IUPM from 0 to 1 cells per million, whereas we used the same prior distribution across the same set of simulations irrespective of outcomes.

When the true IUPM was increased to 1.0, we observed that both QVOA-NGSA and IUPMBayes tended to have lower relative errors than IUPMStats; the difference was highly significant for IUPMBayes (Wilcoxon signed rank test, $P=2.84\times 10^{-7}$; Fig. 4). Furthermore, the number of ‘all-positive’ cases that resulted in $\hat{\lambda }=\infty$ estimates from either IUPMStats or QVOA-NGSA became more frequent with decreasing sample size. For instance, IUPMStats was unable to generate a meaningful estimate of IUPM for 46% of replicate simulations with 2 wells. Even though QVOA-NGSA utilized sequence variation to ameliorate this effect, if any of the variants appeared in all replicate wells, then its maximum likelihood estimator similarly resulted in an infinite value. This effect became severe when we increased the true IUPM to 5.0; IUPMStats yielded valid estimates of IUPM in only 2% of replicates irrespective of sample size, and QVOA-NGSA produced estimates for no more than 12% (Fig. 4). In contrast, IUPMBayes was surprisingly robust to reductions in sample size, with relative errors increasingly only slightly with decreasing numbers of wells when the true IUPM was 1.0 or greater.

We also observed that the 95% confidence intervals generated by the IUPMStats method tend to be slightly broader than the analogous Bayesian credibility intervals we obtained from the empirical 95% intervals. For instance, when the true IUPM was 1 and we simulated four replicate wells, there were five possible outcomes ranging from zero to 4 positives. The IUPM estimates and confidence/credibility intervals obtained by IUPMStats and IUPMBayes for the respective outcomes are summarized in Table 1. The most frequent outcome was that 3 of 4 wells were positive: for this outcome, the IUPMStats estimate was 1.39 with a 95% CI from 0.41 to 4.72, while the median IUPMBayes estimate was 1.36 with a narrower 95% CI from 0.52 to 2.87. Note that these intervals describe the confidence within a single experiment, as opposed to the variance in estimates across replicate experiments. Some of this difference could be attributed to using a prior distribution on $\lambda$. However, we obtained similar results in the simulation experiments with duplicate serial fivefold dilutions and a uniform prior on $\lambda$ (Fig. 2). For example, when the IUPM was set to 1, the most frequent outcome was a single positive well with $10^6$ cells; the 95% C.I. around the IUPMStats estimate of 0.51 was 0.07 to 3.70, while the median IUPMBayes estimate (assuming five variants with frequency ratio 8:4:2:1:1) was 0.91 with a 95% C.I. = (0.14, 3.06). The next most frequent outcome was two positive wells with $10^6$ cells, and the respective results for IUPMStats and IUPMBayes were 1.61 (0.34, 7.51) and 1.91 (0.53, 4.63). Thus, the incorporation of additional information from the presence or absence of specific variants in the QVOA augments our precision in estimating $\lambda$.

### Computing time

Since we employ Bayesian sampling to estimate the model parameters, including IUPM, our method is more time-consuming than methods based on maximum likelihood estimators such as IUPMStats. We evaluated the time required to generate IUPM estimates by measuring run times for IUPMBayes on 10 replicate data sets simulated under the first set of conditions (previous section). On a single core of an Intel E5-1620v2 processor, it required an average of 358.8 seconds (roughly six minutes) to run a chain sample for one million steps, which we have found to be adequate for convergence under a variety of simulated conditions. In contrast, our implementation of IUPMStats in *R* used only 208.3 microseconds on average to process the same data in the same computing environment.

### Empirical data

We applied both the IUPMStats and IUPMBayes methods to actual experimental results derived from two patients (106 and 111). These patients had been selected from a larger study in progress because the overall sequence diversity in viral outgrowth wells derived from the latent reservoirs of the respective individuals were characterized by either very high (106) or very low (111) levels of clonality. We use the term ‘clonality’ to refer to the scenario where the virus population is dominated by a variant or variants that are repeated, such that most sequences in a random sample will be identical or have another well containing that same variant. Based on this prior information, we adjusted the hyperparameters of the Dirichlet prior distribution to $\alpha _1=10, \alpha _{i\ne 1}=1$ for patient 106 and $\alpha _{i}=1$ for patient 111. We assessed the sensitivity of our results to these prior distributions in Additional file 2: Fig. S2. The dilution series for allocating numbers of cells per well was the same as experimental design 1 ($10^6$, $2\times 10^5$, $4\times 10^4$, 8000, 1600, 320). However, the number of wells carrying $10^6$ cells was varied with 8 wells for patient 106 and 18 wells for patient 111.

We generated ML estimates under the SHP model using both our implementation of this method in *R* and the online calculator at http://silicianolab.johnshopkins.edu. These experimental data and results are summarized in Table 2. Our implementation of SHP model estimation in *R* produced numerically identical estimates to the online calculator for both patients. The IUPMStats estimate was significantly higher for patient 106 (8.2 per million) than 111 (1.6 per million). Additionally, we observed fewer distinct sequence variants in well samples from patient 106 than 111 for both the *gp41* and *pol* regions.

**Fig. 5:**
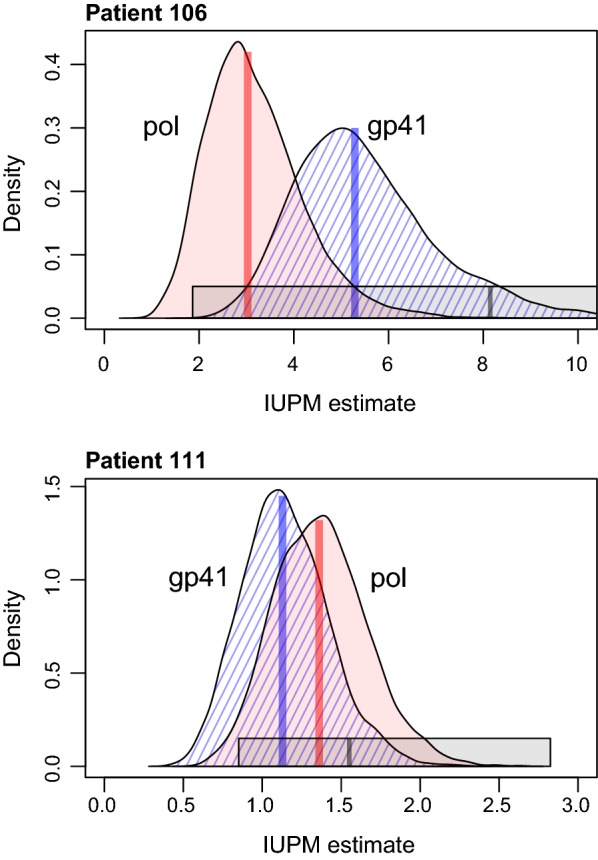
Summary of IUPM estimates obtained by a Bayesian analysis of experimental data from subjects 106 (high clonality) and 111 (low clonality). IUPM was estimated separately using sequence data obtained for HIV regions *pol* (red) and *gp41* (blue, hatched). Three replicate chains were combined for each patient and gene after assessing that the chains had converged to the posterior distribution over the IUPM parameter. Median estimates are indicated by vertical line segments within each density plot. Grey bars represent the maximum likelihood estimate and 95% confidence interval obtained by IUPMStats [17]. In the case of patient 106, the upper confidence limit from IUPMStats extends to 35.6 cells per million

Results from our Bayesian analysis of the same data sets under the MTP model are summarized in Fig. 5. To facilitate evaluation of convergence, we ran three replicate Markov chain samples per data set with different initial parameters. We found that for both patients and genes, the median posterior estimates of IUPM were substantially lower than the ML estimates obtained under the SHP model. For patient 106, the median estimates were 5.28 (95% C.I., 3.19, 9.26) per million for *gp41* and 3.03 (1.60, 5.54) for *pol*. This difference was driven in part by the occurrence of the predominant *gp41* variant in all eight wells carrying $10^6$ cells, whereas the predominant *pol* variant occurred in only seven of these eight. Combining these estimates, we predict that the IUPM in patient 106 was about 3.8 infected cells per million—substantially lower than the IUPMStats estimate, but within its 95% confidence interval (Table 2). The discrepancies between these estimates was consistent with the levels of absolute error we observed with simulated data. In contrast, we obtained more concordant IUPMBayes estimates between gene regions for patient 111; the median posterior estimates were 1.13 (95% C.I., 0.66, 1.74) per million for *gp41* and 1.36 (0.85, 2.00) for *pol*, respectively.

## Discussion

Maximum likelihood (ML) is a powerful technique for estimating the rate parameter of the single-hit Poisson model [19, 20]. However, there can be issues with ML estimation that are exacerbated with small sample sizes. First, the single-hit Poisson estimator becomes infinite when all the wells are positive [21]. We have demonstrated here that this issue can even affect estimators that partition the outcomes by sequence variants, i.e., QVOA-NGSA [7]. Methods that reject data sets where all wells are positive may systematically underestimate the IUPM when the actual value is high, because the investigator is required to perform additional experiments until one or more negative outcomes are obtained. However this scenario is unlikely when the experimental design utilizes a wide-ranging dilution series and can also be mitigated by increasing the number of replicate wells. Second, ML estimators based on discrete outcomes can take only a finite number of values, which we have shown can limit the estimator’s accuracy when the number of informative observations is small (Fig. 3). Both issues are exacerbated by reducing the number of wells used in the experiment. On the other hand, there are significant practical benefits to reducing the number of replicate wells required to estimate the IUPM, not only because the assay requires weeks of culturing cells, but also because scaling up these experiments to large numbers of samples is necessary to detect statistical associations between the IUPM and clinical variables [9, 22].

Our results support the concept of applying Bayesian methods to sequence diversity to overcome the limitations of maximum likelihood for small samples (fewer wells). Although previous studies have implemented Bayesian methods to parameterize the single-hit Poisson model [23], we are not aware of another study taking this approach to incorporate sequence information for estimating the number of infectious units. Additionally, the Bayesian approach designed here could be adapted to more properly incorporate viral sequence data in other LVR assays that estimate the size of inducible or intact proviral HIV infected cell populations, and thereby improve their accuracy and usefulness in HIV cure studies [24, 25]. Using simulations, we have demonstrated that our Bayesian method becomes more accurate than current methods as the true IUPM increases above 1, and that this advantage is greater with increasing sequence diversity in the latent HIV reservoir (Fig. 2). The emerging consensus from empirical evidence is that, on average, slightly less than one in a million resting CD4+ T cells contain replication competent latent HIV. If actual IUPM values tended to be tightly clustered around this expectation, then there would be limited use in incorporating sequence variation for quantifying the latent reservoir. However, empirical evidence indicates that there is substantial variability in IUPM around the mean. For example, Crooks et al. [9] recently reported estimates from 37 patients that ranged by more than two orders of magnitude around a mean of 0.42 per million (about 0.02 to 20 IUPM). Similarly, Eriksson et al. [22] reported a geometric mean of 0.64 IUPM among 30 patients on suppressive therapy with a range also spanning about two orders of magnitude. Therefore it is reasonable to expect a substantial fraction ($\sim$10%) of individuals to have an IUPM that is measurably greater than 1.

A core assumption of our method is that detecting more than one sequence variant in a culture well indicates the presence of at least that number of latently infected cells. It is possible that a cell is multiply infected with two or more integrated HIV DNA variants [26]. This scenario would cause our method to overestimate the IUPM. However, recent work determined that over 85% of infected CD4+ T cells in peripheral blood carried only a single integrated HIV DNA molecule [27], and the majority of these proviruses are defective [4]—thus, the generally low prevalence of replication competent virus in the LVR (see above) supports this simplifying assumption of the model. In addition, our method assumes that any variant that is present in a particular well will be detected by next-generation sequencing, irrespective of its frequency in the initial sample population. In other words, we assume that viral outgrowth is completely efficient; the other methods for analyzing QVOA data require this same assumption irrespective of genetic variation.

The considerable depth at which next-generation sequencing samples templates from the outgrowth population makes it unlikely that variants present in the well are unobserved. However, the rapid evolution of HIV-1 means there may be an unknown number of rare genetic variants in the LVR that fail to become sampled from the source population into any replicate wells of the QVOA experiment, immediately precluding these variants from outgrowth and sequencing. In fitting the multi-hit Poisson model, we have out of necessity restricted the model to the observed variants in the experiment by marginalizing out the number of unobserved variants, $M^*$, which is exceedingly difficult to estimate from these data. This raises a potential issue in that selecting the prior distribution on variant frequencies, *P*(*f*|*M*), becomes directly influenced by the number of observed variants in the data. Strict adherence to Bayesian principles discourages this repeated use of the data, but there is growing recognition that such ‘data-dependent priors’ are routinely used for empirical Bayesian inference and can retain statistically desirable properties, e.g., [28]. For example, IUPMStats employs a data-dependent prior that is a uniform distribution over the interval $\lambda _m=(0,1)$ if all wells in the QVOA experiment are negative, and $\lambda _m=(0, \infty )$ otherwise. There are fully hierarchical Bayesian methods available to accommodate the uncertainty in $M^*$, such as the Dirichlet process prior, which describes a distribution over the different Dirichlet prior distributions for varying numbers of variants. However, MCMC sampling under the Dirichlet process prior is not trivial to implement and can be slow to converge, in part because the number of prior distributions is infinite [29]. Furthermore, we are pessimistic that the presence-absence matrix derived from sequencing QVOA experiments can sustain such an expansion of the model parameter space.

Although our method was developed for the specific purpose of measuring the latent HIV reservoir, it can be adapted to other applications of limiting dilution assays where the target has genetic variation. For example, limiting dilution has been used to measure the frequency of reactivation in cells latently infected with human cytomegalovirus [30]. It could also be adapted for the quantitation of nucleic acids by dilution and amplification-based assays, which have been developed for viruses such as hepatitis C virus [31], and modernized with high-throughput technologies that rely on the same underlying statistical model, such as digital PCR [32]. Finally, limiting dilution and the single-hit Poisson model have historically been deployed in the study of immunocompetent cell populations [19] and continues to play a fundamental role in ongoing studies of the diverse cellular subsets of the immune response [33].

## Conclusions

The existence of the latent viral reservoir is a key barrier to curing HIV-1 despite highly effective drug treatments. In this study, we demonstrate that sequencing individual viruses from the positive wells of a viral outgrowth assay can provide a more robust and accurate measure of the latent reservoir. Furthermore, this approach can facilitate scaling up the assay to large numbers of samples by reducing the number of wells necessary to measure the latent viral reservoir.

## Methods

### Models

To facilitate batch processing on simulated and experimental data sets, we re-implemented the SHP model in the *R* programming language. For brevity, we will use the notation $\lambda _m=\lambda \times 10^6$ for IUPM. We obtained a maximum likelihood estimate ($\hat{\lambda }_m$) given equation (2) using Brent’s root-finding method [34] with a lower bound of $\lambda _m>0$ and an arbitrary upper bound (default $\lambda _m<10^3$). Further, we confirmed that our implementation yielded results consistent with the web-based IUPMStats calculator (http://silicianolab.johnshopkins.edu/, version 1.0). We estimated the 95% confidence interval from the likelihood function using numerical root-finding [34] to solve the equality $L(\lambda _m) / L(\hat{\lambda }_m) = 0.147$ on the intervals $\lambda _m=(10^{-3}, \hat{\lambda }_m)$ and $\lambda _m=(\hat{\lambda }_m, 10^{3})$; this approach assumes the sample size is sufficiently large that the log-likelihood ratio can be approximated by the $\chi ^2$-distribution. When the data comprise entirely of negative wells, the MLE for $\lambda$ in the SHP model is zero. In this case, the IUPMStats calculator substitutes the posterior median estimate given a uniform prior distribution over the interval $\lambda _m=(0,1)$ [17].

Suppose that an experiment has *K* wells and *M* sequence variants. Let $v_{k,i}=\{0,1\}$ be the binomial outcome for the presence or absence of the *i*-th sequence variant in well *k*. Let $f_i$ be the frequency of the *i*-th variant in the population of infected cells in the undiluted QVOA wells. The likelihood that at least one cell in a particular well was infected with the *i*-th variant is described by combining the Bernoulli and Poisson distributions:

3 $$\begin{aligned} L(v_{k,i}| \lambda , f_i) = \left( 1-\exp (-f_i n_k \lambda )\right) ^{v_{k,i}} \exp (-f_i n_k \lambda )^{1-v_{k,i}} \end{aligned}$$

where $f_i n_k \lambda$ is the expected number of cells infected by variant *i* in well *k*. Following standard practice such as the single-hit Poisson model [17], we assume that $n_k$ are known without error. Equation (3) is similar to equation (2), except that (3) accommodates variation in the relative frequencies of sequence variants in the infected cell population. The likelihood for the data set is calculated by taking the product across all *K* wells and *M* variants:

4 $$\begin{aligned} {\mathbf{L}}({\mathbf{v}}|\lambda , f, M) = \prod _{k=1}^{K}\prod _{i=1}^{M} L(v_{k,i}|\lambda , f_i) \end{aligned}$$

where ${\mathbf{v}}$ is a binary $K\times M$ matrix with entries $\{v_{k,i}\}$. This model is similar to the two-target Poisson models used in immunology to accommodate subsets of cells with different frequencies [35]—accordingly, we will refer to equation (4) as the multi-target Poisson (MTP) model.

HIV-1 can become highly diverse within hosts [36] and not all variants in the population are necessarily observed in the experiment, especially those at very low frequencies. The number of unobserved variants, which we denote by $M^*$, is an uncertain quantity and not feasible to estimate from the QVOA data. We will express this by expanding equation (4) as follows:

5 $$\begin{aligned} {\mathbf{L}}({\mathbf{v}} | \lambda , f, M, M^*) = \prod _{k=1}^{K}\left( \prod _{i=1}^{M} L(v_{k,i}|\lambda , f_i) \prod _{i=M+1}^{M+M^*} L(0|\lambda , f_i) \right) \end{aligned}$$

where *M* now refers to the observed number of variants instead of the total number. Our objective is use this likelihood to estimate $\lambda$ where *f*, *M* and $M^*$ are nuisance parameters. The viral outgrowth intrinsic to this assay precludes the use of next-generation sequencing to directly estimate variant frequencies *f*, which become obscured by the highly stochastic nature of viral amplification. Thus, we used Bayesian inference to estimate the posterior distribution of $\lambda$ while accommodating the uncertainty in the other model parameters. Let the total number of variants be represented by an unspecified prior distribution $P(M,M^*)$ that ranges from 1 to $\infty$. We used the standard Dirichlet prior distribution for the variant frequencies *f* given $M+M^*$ variants:

6 $$\begin{aligned} P(f| M, M^*)=\Gamma \left( \sum\nolimits_{i=1}^{M+M^*}\alpha _i \right) \prod _{i=1}^{M+M^*} \frac{f_i^{\alpha _i-1}}{\Gamma (\alpha _i)} \end{aligned}$$

where the hyperparameters $\alpha _i$ can be interpreted as the respective counts of the variants in a hypothetical sample a priori. By default, we set $\alpha _i=1$ for all *i* to obtain a flat uninformative prior distribution over all possible values of *f*. We evaluated different prior distributions for $\lambda$: first, we omitted $\lambda$ from our calculation of prior probability, which was equivalent to an unbounded uniform prior; second, we used a gamma distribution with shape parameter $\alpha =2$ and rate parameter $\beta =1$, which yields a median of 1.68 and 95% interval of (0.24, 5.6). The posterior probability of the multi-hit Poisson model can thereby be written:

7 $$\begin{aligned} P(\lambda , f, M, M^* | {\mathbf{v}}) \propto {\mathbf{L}}({\mathbf{v}} | \lambda , f, M, M^*) P(\lambda ) P(f| M, M^*) P(M, M^*) \end{aligned}$$

Rather than sample over possible values of $M^*$ for which we have no information, we exclude the unobserved variants from the data matrix and marginalize out this parameter:

8 $$\begin{aligned} P(\lambda , f | {\mathbf{v}}^+)\propto & {} \sum _{M^*=0}^{\infty } {\mathbf{L}}({\mathbf{v}}^+ | \lambda , f, M=M_{{\mathbf{v}}^{+}}) P(\lambda ) P(f|M=M_{{\mathbf{v}}^{+}}) P(M^*|M=M_{{\mathbf{v}}^{+}}) \nonumber \\\propto & {} \left( \prod _{k=1}^{K} \prod _{i=1}^{M_{{\mathbf{v}}^+}} L(v_{k,i}|\lambda , f_i) \right) P(\lambda ) P(f|M=M_{{\mathbf{v}}^+}) \end{aligned}$$

where ${\mathbf{v}}^+$ excludes all $M^*$ columns in ${\mathbf{v}}$ where $\sum _{k=0}^{K}v_{k,\cdot }=0$, *f* is renormalized so that $\sum _{i=1}^{M} f_i = 1$, and ${\mathbf{L}}({\mathbf{v}}^+ | \lambda , f, M=M_{{\mathbf{v}}^{+}})$ is the likelihood conditioned on observing $M_{{\mathbf{v}}^+}$ variants in the data. Since this conditional likelihood and the priors $P(\lambda )$ and *P*(*f*|*M*) are assumed to be independent of $M^*$, they can be moved outside of the sum, which becomes a constant term that drops out of the proportionality relation. Note that in equation (8), *M* is set to the observed number of variants in the data ($M_{{\mathbf{v}}^+}$) instead of being inferred as a model parameter. We will revisit these assumptions in the “Discussion” section.

**Table 1:** Comparison of estimates and 95% confidence/credibility intervals for IUPMStats and IUPMBayes

Positives	*n*	IUPMStats			IUPMBayes		
		Estimate	Lower 95%	Upper 95%	Median	Lower 95%	Upper 95%
0	3	0.17*	0	0.75	0.34	0.05	1.10
1	16	0.29	0.04	2.06	0.61	0.15	1.68
2	28	0.69	0.17	2.85	1.04	0.36	2.41
3	37	1.39	0.41	4.72	1.36	0.52	2.87
4	16	∞	Undefined		1.85	0.80	3.63

We summarized the results of each method on 100 simulations of 4 replicate wells with $10^6$ cells each, where the true IUPM was set to 1.0 with five sequence variants of equal frequency (1:1:1:1:1). We obtained results for the IUPMStats method directly from the online calculator. Entries for IUPMBayes were averaged across replicate simulations for each number of positives. *IUPMStats uses a median posterior estimate instead of the maximum likelihood estimate (0) when none of the wells are positive

Markov chain Monte Carlo (MCMC) samples were propagated over the model parameters $\lambda$ and *f* by Metropolis–Hastings sampling. The chains were arbitrarily initialized at the parameter values $\lambda _m=5$ and $f_i=1/M$ for all *i*. We used a Gaussian proposal function for $\lambda$ with a mean centred at the current value of $\lambda$ and standard deviation $\sigma =0.1$, i.e., $\lambda '\sim \mathcal {N}(\lambda , 0.1)$. Because $\lambda$ has a lower bound of zero, we took the absolute value of the proposed $\lambda$; the resulting proposal distributions retain the important property that $q(x|x')=q(x'|x)$. We proposed new variant frequencies from the uniform distributions $(f_i-\delta , f_i+\delta )$ where $\delta$ was set to 0.005, and normalized the results so that $\sum _{i=1}^{M} f_i=1$. Again, these proposal distributions were reflected at zero so that all $f_i>0$. The MCMC sampler was implemented in *R*.

### Method validation

To evaluate the performance of different methods to estimate IUPM, we simulated virus outgrowth data sets in *R* by first sampling the number of infected cells per well, $Y\sim {\text{Poisson}}(n\lambda )$. Next, the infected cells were partitioned among *N* sequence variants by drawing from the multinomial distribution defined by the frequency vector $f=(f_1, \ldots , f_N)$ (note we changed notation to avoid confusing this parameter with *M* and $M^*$). If a sequence variant was completely absent from the simulated data, then we omitted the corresponding column of zero counts from the data set (subsetting ${\mathbf{v}}$ to ${\mathbf{v}}^{+}$ as above) as a necessary part of calculating the conditional likelihood (Eq. 8). Put another way, if a particular variant was not observed any wells, then we assumed that there was no way of inferring that variant’s presence in the source population.

To systematically evaluate the impact of different simulation conditions, we conducted simulations for all combinations of rate parameters $\lambda _m=\{0.2, 1, 5, 25\,{\text{per million cells}}\}$ and variant frequencies $f=\{(0.5, 0.5), (0.2, 0.2, 0.2, 0.2, 0.2), (0.5, 0.25, 0.125, 0.0625, 0.0625)$}, which we denote more concisely as ratios 1:1, 1:1:1:1:1 and 8:4:2:1:1. For each combination of model parameters, we generated 100 replicate data sets and applied the maximum likelihood and Bayesian methods to estimate the IUPM. Each MCMC sample was propagated for $10^6$ steps with the first $10^5$ steps discarded as burn-in; the remaining chain sample was thinned at regular intervals of 1000 steps. We chose these settings based on our analysis of the convergence behaviour and autocorrelation of preliminary runs (Additional file 3: Fig. S3). To reduce computing time, all simulations and model analyses were run on an Intel Xeon-based computing cluster using the *R* module *parallel*. The concordance of model estimates and actual values was visually assessed with density plots and quantified by the relative error, which was calculated as the absolute difference between the actual and true values, normalized by the true value.

**Table 2:** Summary of outgrowth assay results for two patients

Patient	Positive wells (total wells)						IUPMStats	# variants
	$\mathsf{10^6}$	$\mathsf{2\times 10^5}$	$\mathsf{4\times 10^4}$	$\mathsf{8000}$	$\mathsf{1600}$	$\mathsf{320}$ cells	(95% CI)	(*gp41*, *pol*)
106	8 (8)	2 (2)	0 (2)	0 (2)	0 (2)	0 (2)	8.148	10, 4
							(1.863, 35.635)	
111	13 (16)	0 (2)	0 (2)	0 (2)	0 (2)	0 (2)	1.551	13, 20
							(0.851, 2.825)	

For each dilution (number of cells per well), we report the number of positive wells (total number of wells). IUPM estimates and 95% confidence intervals (CIs) under the single-hit Poisson model were generated using the JavaScript calculator at http://silicianolab.johnshopkins.edu (last access date: June 23, 2017). The final column reports the number of sequence variants that were observed in regions within HIV *gp41* and *pol*, respectively, when HIV RNA was extracted, amplified and sequenced from the positive wells

### Data collection and processing

Actual HIV-1 sequence data was obtained from QVOA previously performed on a population of HIV-individuals recruited through the Rakai Health Science Program in Uganda, originally enrolled to examine the size of the HIV-1 LVR [37]. Participants were HIV-1-infected adults ($\ge$18 years) on ART who had been virally suppressed for $>1$ year at the time of enrollment (two historical plasma HIV-1 RNA measurements $<\,40$ copies/mL, obtained 10–18 months apart with no intervening detectable viral load result).

p24 positive viral outgrowth wells derived from QVOA of rCD4 T cells from these Ugandan subjects were collected and stored at $-\,80^{\circ }$C. HIV viral RNA was extracted from 140 μL of outgrowth media, amplified with a directed nested RT-PCR assay for both *gp41* ($\sim$350 bp) and *pol* ($\sim$530 bp). Paired-end libraries (2 × 300 bases) were generated from the amplicons and barcoded as previously described [38] for sequencing on an Illumina MiSeq system. Adapter sequences were trimmed from the short read data using *cutadapt* (version 1.8) [39] and mate pairs were merged using *AdapterRemoval* [40] with a minimum read overlap of 15 nucleotides. Merged sequences containing ambiguous nucleotide calls were filtered out and clustered using USEARCH (ver.10) with an identity threshold of 0.98 to generate a consensus sequence. The number of times each sequence variant was observed in the data was recorded in the sequence label. Any sequence variant representing < 2.5% of the total number of sequences was excluded from further analysis [7]. The remaining sequence variants were manually screened for large internal deletions.

Wells containing three or more sequence variants (that were greater than 2.5% of the total number of sequences) were manually examined for evidence of recombination as a result of ex vivo recombination in the well or crossover events during library preparation and sequencing, by visually inspecting a Highlighter plot [41] generated from all variant consensus sequences. Any potential recombinant was removed from the data unless the same sequence appeared in another well from the same patient, which implied that the sequence represented a true recombination event within that patient’s virus population (Additional file 4: Fig. S4). A multiple sequence alignment (*MAFFT* ver. 7) was generated from the resulting set of sequence variants and used to reconstruct a phylogenetic tree using the implementation of the neighbor-joining method [42] in *R* for visual inspection. Each unique sequence variant was assigned a unique number and the presence/absence of the variant was recorded for each well.

### Empirical data analysis

We used the SHP (IUPMStats) and Bayesian MTP (IUPMBayes) methods to analyze sequence data from viral outgrowth assays for samples derived from two individuals (denoted 106 and 111) from the Ugandan study cohort. There were two sequence data sets per patient corresponding to the *pol* and *gp41* amplicons, for a total of four data sets. The clonal prediction scores, which quantify the proportions (maximum 100) of unique sequence variants that would be correctly identified by a particular subsequence [43], were 90 ($\pm\,14$) and 83 ($\pm\,26$) for these regions due in part to the relatively small amplicon size required for targeted NGS of the Illumina system. Since we were analyzing a much smaller number of data sets than the simulation study, we ran three replicate chain samples per data set to evaluate convergence. Each chain was initialized with random parameter values; specifically, $\lambda _m$ was drawn from a uniform distribution spanning 1 to 10, and $f$ was drawn from a flat Dirichlet distribution ($\alpha _i=1\;\forall \; i$). We ran each chain sample for $10^7$ steps with the first $5\times 10^5$ steps discarded as the burn-in interval. Chain sample states (log posterior probability and model parameter values) were written to log files at regular intervals of 5000 steps. Convergence was assessed using the Gelman-Rubin diagnostic implemented in the *R* package *coda*.

## Additional files

**Additional file 1. Fig. S1.** Relative errors in IUPM estimates for simulated data with highly skewed variant frequencies. The composition of this figure is similar to Fig. 2.

**Additional file 2. Fig. S2.** Effect of misspecified prior distributions on posterior estimates of IUPM from real data sets. The filled curves represent the same posterior distributions as in Fig. 5, where the prior distributions on variant frequencies were set to *α* = {10, 1,… , 1} for patient 106 and *α* = {1,…, 1} for patient 111. The dashed curves represent the posterior distributions obtained when these priors were swapped between the patient data sets. These results illustrate that misspecification of the prior distribution on variant frequencies can have a measurable effect on posterior estimates of IUPM where the underlying variant frequencies are skewed toward a single common variant (patient 106). However when the virus population has low clonality and there is a mixture of positive and negative wells at the lowest dilution of the QVOA (patient 111, see Table 2), the posterior estimates are more robust to the prior settings.

**Additional file 3. Fig. S3.** Summary of convergence properties for MCMC sampling. The plots display results for three replicate Markov chain samples (black, red, blue) on the same simulated data set, where there were eight wells with 106 cells, four wells with 4 × 104 cells, and four wells with 320 cells; the true IUPM was set to *λ* = 1; and the variant frequencies were set to **f** = {0.5, 0.25, 0.125, 0.0625, 0.0625}. (left) Decay of autocorrelation with increasing lag between samples in the Markov chain. Throughout the study, we thinned chain samples at a lag of 1000 steps. (right) Traces of posterior probability for the first 10,000 steps of the three replicate Markov chains, which corresponds to the length of the burn-in period used in this study. Based on these results, the rate of approach to the posterior distribution was fairly rapid: on the order of 1000 steps.

**Additional file 4. Fig. S4.** Prominent species identification and elimination of potential outgrowth derived recombinant sequences. **a** All Pol derived consensus sequences with amplicon totals greater than 0.02% of the total amplicon number for well 5M4 (1,000,000 rCD4+ cells plated) from patient 111 were aligned and viewed in a Neighbor-Joining (NJ) phylogenetic tree. Prominent species were defined as those with amplicon totals > 2.5% of the total amplicon read number for well 5M4 and are indicated with red arrows. **b** Since the number of prominent species in well 5M4 were ≥ 3 the prominent sequences were aligned and viewed in a highlighter plot. The probable outgrowth derived recombinant sequence is indicated with a red X and was removed from the analysis (probable recombination area highlighted in red box). **c** All Pol prominent outgrowth sequences for patient 111 were viewed in a NJ tree and variants assigned based on clonality (well 5M4 indicated with red arrows).

## References

[1] Churchill MJ, Deeks SG, Margolis DM, Siliciano RF, Swanstrom R (2016). HIV reservoirs: what, where and how to target them. Nat Rev Microbiol.

[2] Harrigan PR, Whaley M, Montaner JS (1999). Rate of HIV-1 RNA rebound upon stopping antiretroviral therapy. AIDS.

[3] Bruner KM, Hosmane NN, Siliciano RF (2015). Towards an HIV-1 cure: measuring the latent reservoir. Trends Microbiol.

[4] Ho YC, Shan L, Hosmane NN, Wang J, Laskey SB, Rosenbloom DI (2013). Replication-competent noninduced proviruses in the latent reservoir increase barrier to HIV-1 cure. Cell.

[5] Finzi D, Hermankova M, Pierson T, Carruth LM, Buck C, Chaisson RE (1997). Identification of a reservoir for HIV-1 in patients on highly active antiretroviral therapy. Science.

[6] Laird GM, Rosenbloom DI, Lai J, Siliciano RF, Siliciano JD, Prasad VR, Kalpana GV (2016). Measuring the frequency of latent HIV-1 in resting CD4+ T cells using a limiting dilution coculture assay. HIV Protocols.

[7] Lee SK, Zhou S, Baldoni PL, Spielvogel E, Archin NM, Hudgens MG (2017). Quantification of the latent HIV-1 reservoir using ultra deep sequencing and primer ID in a viral outgrowth assay. J Acquir Immune Defic Syndr.

[8] Finzi D, Blankson J, Siliciano JD, Margolick JB, Chadwick K, Pierson T (1999). Latent infection of CD4+ T cells provides a mechanism for lifelong persistence of HIV-1, even in patients on effective combination therapy. Nat Med.

[9] Crooks AM, Bateson R, Cope AB, Dahl NP, Griggs MK, Kuruc JD (2015). Precise quantitation of the latent HIV-1 reservoir: implications for eradication strategies. J Infect Dis.

[10] Hosmane NN, Kwon KJ, Bruner KM, Capoferri AA, Beg S, Rosenbloom DIS (2017). Proliferation of latently infected CD4(+) T cells carrying replication-competent HIV-1: potential role in latent reservoir dynamics. J Exp Med.

[11] Maldarelli F, Wu X, Su L, Simonetti FR, Shao W, Hill S (2014). HIV latency. Specific HIV integration sites are linked to clonal expansion and persistence of infected cells. Science.

[12] Simonetti FR, Sobolewski MD, Fyne E, Shao W, Spindler J, Hattori J (2016). Clonally expanded CD4+ T cells can produce infectious HIV-1 in vivo. Proc Natl Acad Sci USA.

[13] Chun TW, Carruth L, Finzi D, Shen X, DiGiuseppe JA, Taylor H (1997). Quantification of latent tissue reservoirs and total body viral load in HIV-1 infection. Nature.

[14] Bui JK, Sobolewski MD, Keele BF, Spindler J, Musick A, Wiegand A (2017). Proviruses with identical sequences comprise a large fraction of the replication-competent HIV reservoir. PLoS Pathog.

[15] Lorenzi JCC, Cohen YZ, Cohn LB, Kreider EF, Barton JP, Learn GH (2016). Paired quantitative and qualitative assessment of the replication-competent HIV-1 reservoir and comparison with integrated proviral DNA. Proc Natl Acad Sci USA.

[16] Miller RG, Teh HS, Harley E, Phillips RA (1977). Quantitative studies of the activation of cytotoxic lymphocyte precursor cells. Immunol Rev.

[17] Rosenbloom DI, Elliott O, Hill AL, Henrich TJ, Siliciano JM, Siliciano RF, Oxford University Press (2015). Designing and interpreting limiting dilution assays: general principles and applications to the latent reservoir for human immunodeficiency virus-1. Open Forum. Infect Dis..

[18] Wineberg M, Oppacher F. The underlying similarity of diversity measures used in evolutionary computation. In: Genetic and evolutionary computation—GECCO 2003. Springer; 2003. p. 206–206.

[19] Taswell C (1981). Limiting dilution assays for the determination of immunocompetent cell frequencies. I. Data analysis. J Immunol..

[20] Stallard N, Gravenor MB, Curnow RN (2006). Estimating numbers of infectious units from serial dilution assays. J R Stat Soc C.

[21] Mehrabi Y, Matthews J (1995). Likelihood-based methods for bias reduction in limiting dilution assays. Biometrics.

[22] Eriksson S, Graf EH, Dahl V, Strain MC, Yukl SA, Lysenko ES (2013). Comparative analysis of measures of viral reservoirs in HIV-1 eradication studies. PLoS Pathog.

[23] Mehrabi Y, Matthews JNS (1998). Implementable Bayesian designs for limiting dilution assays. Biometrics.

[24] Yucha RW, Hobbs KS, Hanhauser E, Hogan LE, Nieves W, Ozen MO (2017). High-throughput characterization of HIV-1 reservoir reactivation using a single-cell-in-droplet PCR assay. EBioMedicine.

[25] Sanyal A, Mailliard RB, Rinaldo CR, Ratner D, Ding M, Chen Y (2017). Novel assay reveals a large, inducible, replication-competent HIV-1 reservoir in resting CD4(+) T cells. Nat Med.

[26] Jung A, Maier R, Vartanian JP, Bocharov G, Jung V, Fischer U (2002). Recombination: multiply infected spleen cells in HIV patients. Nature.

[27] Josefsson L, King MS, Makitalo B, Brännström J, Shao W, Maldarelli F (2011). Majority of CD4+ T cells from peripheral blood of HIV-1-infected individuals contain only one HIV DNA molecule. Proc Natl Acad Sci.

[28] Wasserman L (2000). Asymptotic inference for mixture models by using data-dependent priors. J R Stat Soc Ser B (Stat Methodol).

[29] Ghosal S (2010). The Dirichlet process, related priors and posterior asymptotics. Bayesian Nonparametr.

[30] Goodrum F, Jordan CT, Terhune SS, High K, Shenk T (2004). Differential outcomes of human cytomegalovirus infection in primitive hematopoietic cell subpopulations. Blood.

[31] Hawkins A, Davidson F, Simmonds P (1997). Comparison of plasma virus loads among individuals infected with hepatitis C virus (HCV) genotypes 1, 2, and 3 by quantiplex HCV RNA assay versions 1 and 2, Roche Monitor assay, and an in-house limiting dilution method. J Clin Microbiol.

[32] McCaughan F, Dear PH (2010). Single-molecule genomics. J Pathol.

[33] Tubo NJ, Pagán AJ, Taylor JJ, Nelson RW, Linehan JL, Ertelt JM (2013). Single naive CD4+ T cells from a diverse repertoire produce different effector cell types during infection. Cell.

[34] Brent RP (1973). Algorithms for minimization without derivatives.

[35] Bonnefoix T, Bonnefoix P, Verdiel P, Sotto JJ (1996). Fitting limiting dilution experiments with generalized linear models results in a test of the single-hit Poisson assumption. J Immunol Methods.

[36] Shankarappa R, Margolick JB, Gange SJ, Rodrigo AG, Upchurch D, Farzadegan H (1999). Consistent viral evolutionary changes associated with the progression of human immunodeficiency virus type 1 infection. J Virol.

[37] Prodger JL, Lai J, Reynolds SJ, Keruly JC, Moore RD, Kasule J (2017). Reduced frequency of cells latently infected with replication-competent HIV-1 in virally suppressed individuals living in Rakai, Uganda. Clin Infect Dis.

[38] Courtney CR, Mayr L, Nanfack AJ, Banin AN, Tuen M, Pan R (2017). Contrasting antibody responses to intrasubtype superinfection with CRF02\_AG. PLoS ONE.

[39] Martin M (2011). Cutadapt removes adapter sequences from high-throughput sequencing reads. EMBnet.journal..

[40] Lindgreen S (2012). AdapterRemoval: easy cleaning of next-generation sequencing reads. BMC Res Not.

[41] Keele BF, Giorgi EE, Salazar-Gonzalez JF, Decker JM, Pham KT, Salazar MG (2008). Identification and characterization of transmitted and early founder virus envelopes in primary HIV-1 infection. Proc Natl Acad Sci.

[42] Thompson JD, Higgins DG, Gibson TJ (1994). CLUSTAL W: improving the sensitivity of progressive multiple sequence alignment through sequence weighting, position-specific gap penalties and weight matrix choice. Nucleic Acids Res.

[43] Laskey SB, Pohlmeyer CW, Bruner KM, Siliciano RF (2016). Evaluating clonal expansion of HIV-infected cells: optimization of PCR strategies to predict clonality. PLoS Pathog.

