# Supplementary figures and images for: Quantitation of the latent HIV-1 reservoir from the sequence diversity in viral outgrowth assays
Source: Retrovirology. 2018 Jul 5;15:47. doi: 10.1186/s12977-018-0426-1 (PMC6034329; doi:10.1186/s12977-018-0426-1)

Relative error

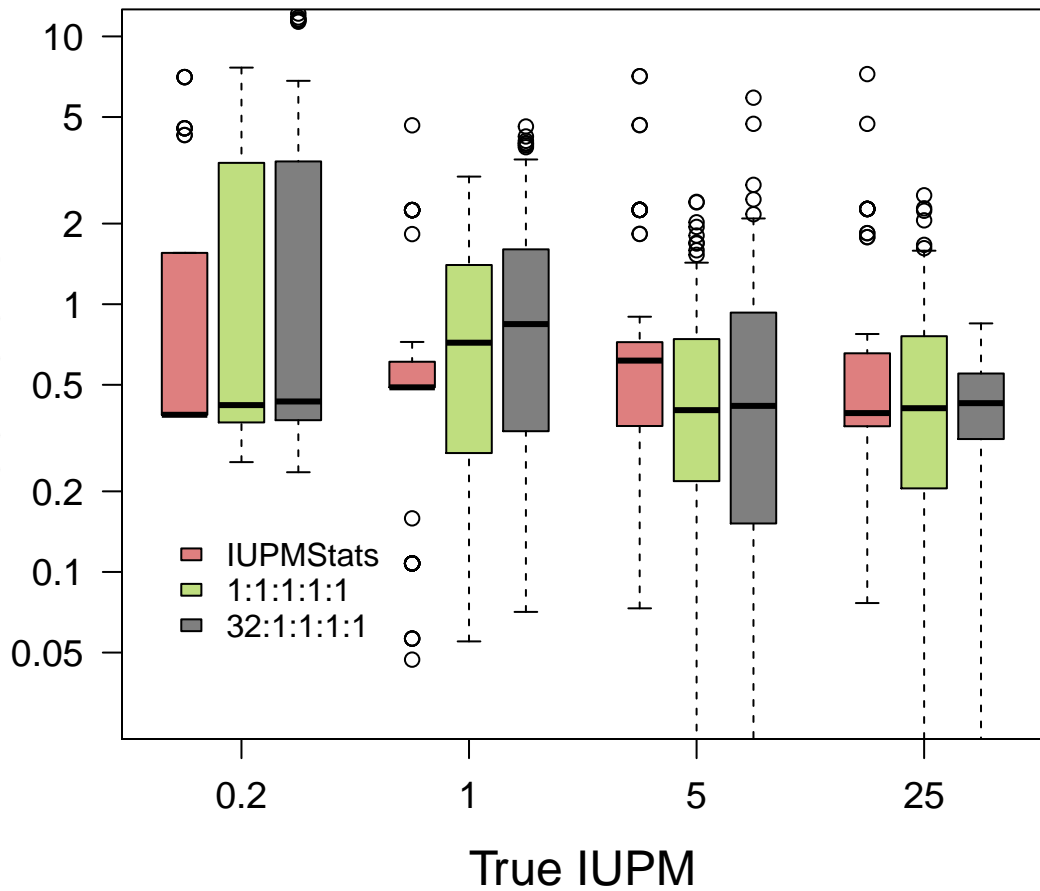

Supplement: Supplementary file 1 — Additional file 1. Fig. S1. Relative errors in IUPM estimates for simulated data with highly skewed variant frequencies. The composition of this figure is similar to Fig. 2. [file 12977_2018_426_MOESM1_ESM.pdf]

**Patient 106**

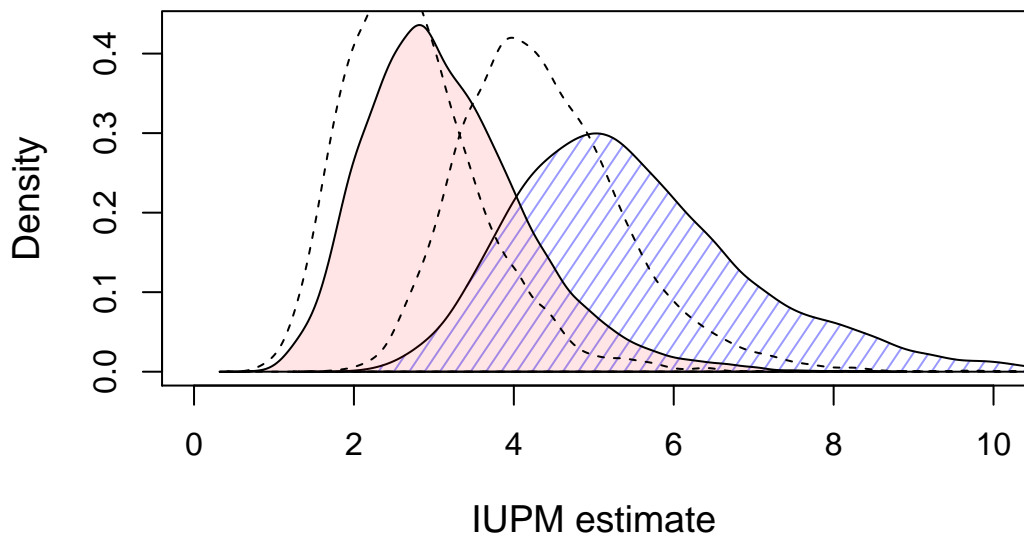

**Patient 111**

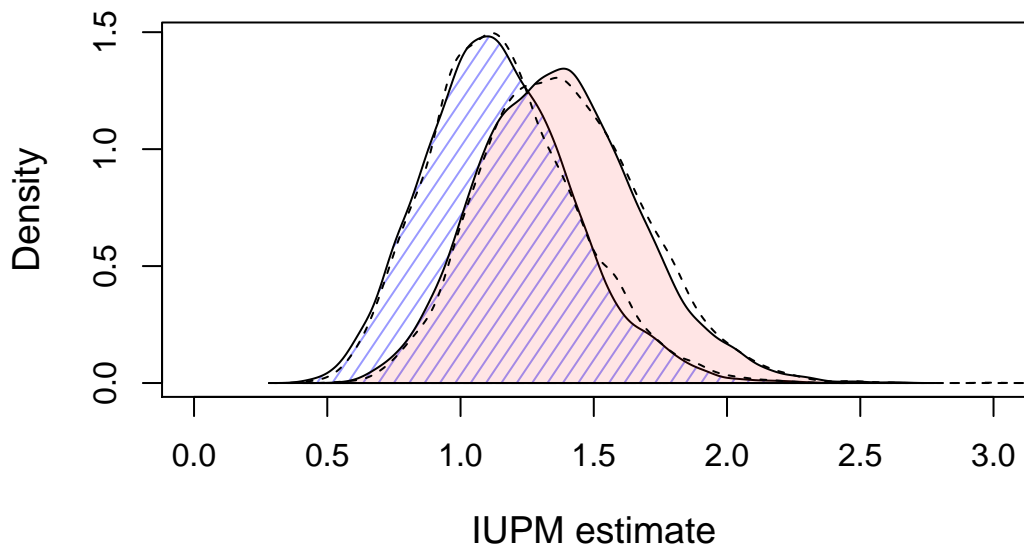

Supplement: Supplementary file 2 — Additional file 2. Fig. S2. Effect of misspecified prior distributions on posterior estimates of IUPM from real data sets. The filled curves represent the same posterior distributions as in Fig. 5, where the prior distributions on variant frequencies were set to α = {10, 1,… , 1} for patient 106 and α = {1,…, 1} for patient 111. The dashed curves represent the posterior distributions obtained when these priors were swapped between the patient data sets. These results illustrate that misspecification of the prior distribution on variant frequencies can have a measurable effect on posterior estimates of IUPM where the underlying variant frequencies are skewed toward a single common variant (patient 106). However when the virus population has low clonality and there is a mixture of positive and negative wells at the lowest dilution of the QVOA (patient 111, see Table 2), the posterior estimates are more robust to the prior settings. [file 12977_2018_426_MOESM2_ESM.pdf]

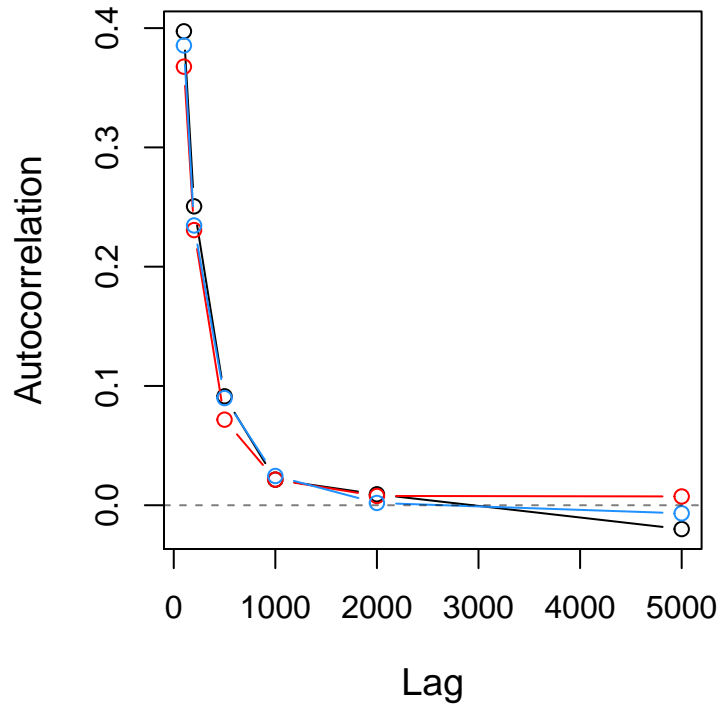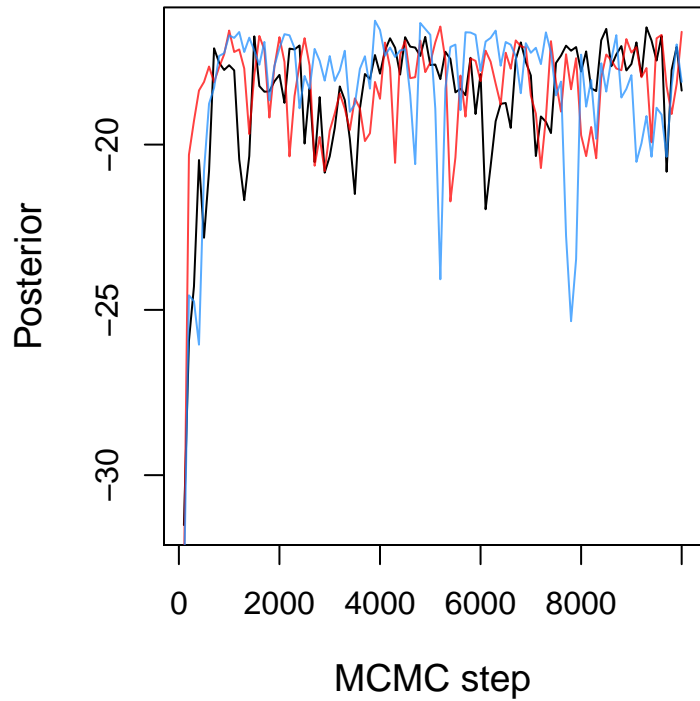

Supplement: Supplementary file 3 — Additional file 3. Fig. S3. Summary of convergence properties for MCMC sampling. The plots display results for three replicate Markov chain samples (black, red, blue) on the same simulated data set, where there were eight wells with 106 cells, four wells with 4 × 10^4^ cells, and four wells with 320 cells; the true IUPM was set to λ = 1; and the variant frequencies were set to f = {0.5, 0.25, 0.125, 0.0625, 0.0625}. (left) Decay of autocorrelation with increasing lag between samples in the Markov chain. Throughout the study, we thinned chain samples at a lag of 1000 steps. (right) Traces of posterior probability for the first 10,000 steps of the three replicate Markov chains, which corresponds to the length of the burn-in period used in this study. Based on these results, the rate of approach to the posterior distribution was fairly rapid: on the order of 1000 steps. [file 12977_2018_426_MOESM3_ESM.pdf]

A)

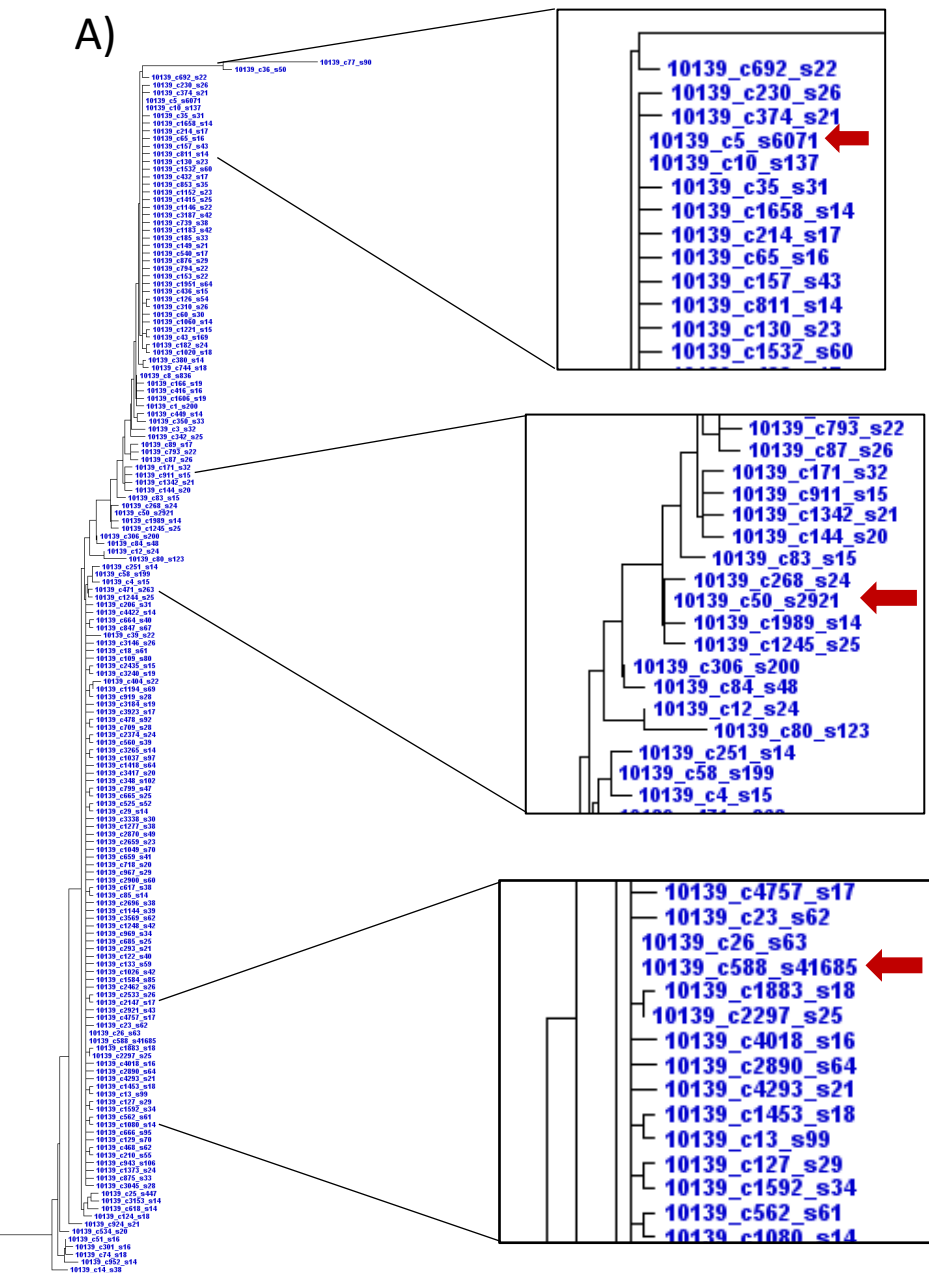

B)

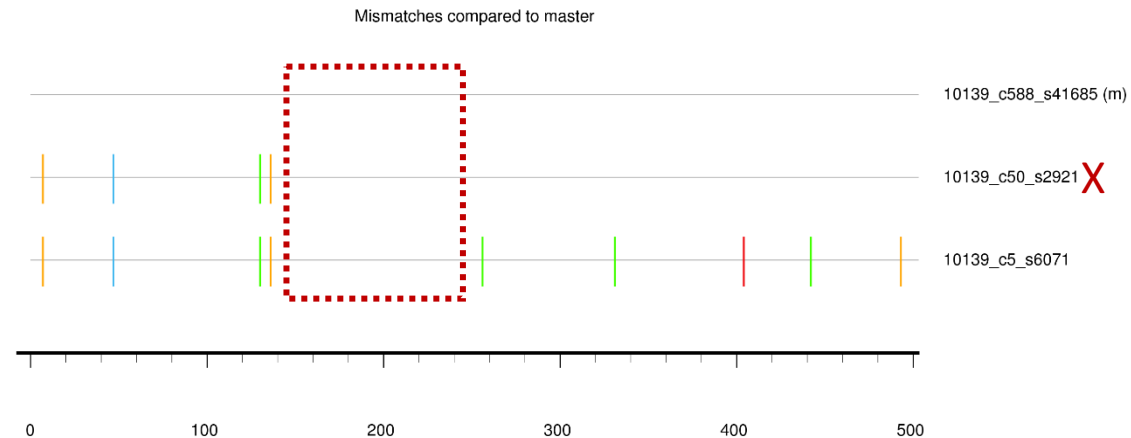

C)

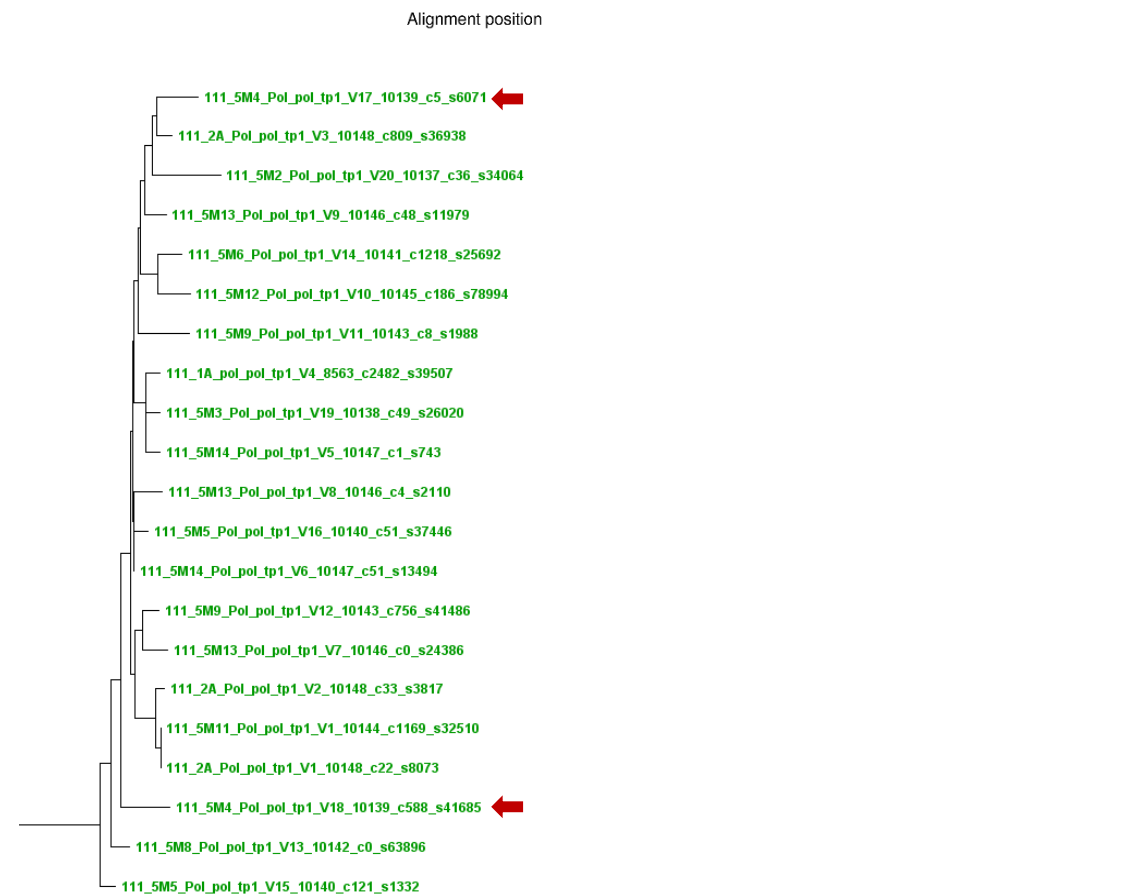

Supplement: Supplementary file 4 — Additional file 4. Fig. S4. Prominent species identification and elimination of potential outgrowth derived recombinant sequences. a All Pol derived consensus sequences with amplicon totals greater than 0.02% of the total amplicon number for well 5M4 (1,000,000 rCD4+ cells plated) from patient 111 were aligned and viewed in a Neighbor-Joining (NJ) phylogenetic tree. Prominent species were defined as those with amplicon totals > 2.5% of the total amplicon read number for well 5M4 and are indicated with red arrows. b Since the number of prominent species in well 5M4 were ≥ 3 the prominent sequences were aligned and viewed in a highlighter plot. The probable outgrowth derived recombinant sequence is indicated with a red X and was removed from the analysis (probable recombination area highlighted in red box). c All Pol prominent outgrowth sequences for patient 111 were viewed in a NJ tree and variants assigned based on clonality (well 5M4 indicated with red arrows). [file 12977_2018_426_MOESM4_ESM.pdf]
